# Microbial Diversity and Activity During the Biodegradation in Seawater of Various Substitutes to Conventional Plastic Cotton Swab Sticks

**DOI:** 10.3389/fmicb.2021.604395

**Published:** 2021-07-15

**Authors:** Justine Jacquin, Nolwenn Callac, Jingguang Cheng, Carolane Giraud, Yonko Gorand, Clement Denoual, Mireille Pujo-Pay, Pascal Conan, Anne-Leila Meistertzheim, Valerie Barbe, Stéphane Bruzaud, Jean-François Ghiglione

**Affiliations:** ^1^CNRS, UMR 7621, Laboratoire d’Océanographie Microbienne, Observatoire Océanologique de Banyuls, Sorbonne Université, Paris, France; ^2^Innovation Plasturgie et Composites, Biopole Clermont Limagne, Saint-Beauzire, France; ^3^CNRS, UMR 9220 ENTROPIE, Ifremer (LEAD-NC), IRD, Univ Nouvelle–Calédonie, Univ La Réunion, Nouméa, New Caledonia; ^4^Plateforme EnRMAT, Laboratoire PROMES, Rembla de la Thermodynamique, Perpignan, France; ^5^UMR CNRS 6027, Institut de Recherche Dupuy de Lôme (IRDL), Université de Bretagne-Sud, Lorient, France; ^6^SAS Plastic@Sea, Observatoire Océanologique de Banyuls, Banyuls-sur-Mer, France; ^7^Génomique Métabolique, Genoscope, Institut François Jacob, CEA, CNRS, Univ Evry, Université Paris-Saclay, Evry, France

**Keywords:** plastisphere, biofouling, plastic biodegradation, single-used plastics, microbial colonization

## Abstract

The European Parliament recently approved a new law banning single-use plastic items for 2021 such as plastic plates, cutlery, straws, cotton swabs, and balloon sticks. Transition to a bioeconomy involves the substitution of these banned products with biodegradable materials. Several materials such as polylactic acid (PLA), polybutylene adipate terephthalate (PBAT), poly(butylene succinate) (PBS), polyhydroxybutyrate-valerate (PHBV), Bioplast, and Mater-Bi could be good candidates to substitute cotton swabs, but their biodegradability needs to be tested under marine conditions. In this study, we described the microbial life growing on these materials, and we evaluated their biodegradability in seawater, compared with controls made of non-biodegradable polypropylene (PP) or biodegradable cellulose. During the first 40 days in seawater, we detected clear changes in bacterial diversity (Illumina sequencing of 16S rRNA gene) and heterotrophic activity (incorporation of ^3^H-leucine) that coincided with the classic succession of initial colonization, growth, and maturation phases of a biofilm. Biodegradability of the cotton swab sticks was then tested during another 94 days under strict diet conditions with the different plastics as sole carbon source. The drastic decrease of the bacterial activity on PP, PLA, and PBS suggested no bacterial attack of these materials, whereas the bacterial activity in PBAT, Bioplast, Mater-Bi, and PHBV presented similar responses to the cellulose positive control. Interestingly, the different bacterial diversity trends observed for biodegradable vs. non-biodegradable plastics allowed to describe potential new candidates involved in the degradation of these materials under marine conditions. This better understanding of the bacterial diversity and activity dynamics during the colonization and biodegradation processes contributes to an expanding baseline to understand plastic biodegradation in marine conditions and provide a foundation for further decisions on the replacement of the banned single-used plastics.

## Introduction

The growing worldwide use of plastics together with waste mismanagement resulted in an estimated release of more than five trillion plastic particles weighing over 250,000 tons afloat in the oceans, resulting in dramatic toxicological effects along the marine trophic chain (Eriksen et al., 2014; Wang et al., 2019). A large majority of plastic items found at sea is made of polyethylene (PE), polypropylene (PP), and polystyrene (PS), which are more likely to float, and polyvinyl chloride (PVC), nylons, and polyethylene terephthalate (PET), which are more likely to sink (Auta et al., 2017).

Government regulations have been recently established to restrict the use of single-use plastics and promote the biodegradable plastic industry. For example, since 2018, at least 127 countries have established regulations against plastic bags (UNEP, 2018). The European Parliament voted to ban many other single-use plastics for 2021, including plates, cutlery, straws, food containers, and expanded polystyrene cups as well as sticks used for cotton swabs and plastic balloons (Europarl, 2019). Biodegradable plastics have become the focus of recent researches in order to replace conventional materials made of PE, PP, PS, PVC, PET, and other high-molecular compounds. Today, commercially available biodegradable materials are of petrochemical origin, such as poly(butylene succinate) (PBS) and polybutylene adipate terephthalate (PBAT; brand name Ecoflex^®^), or are bio-based such as polylactic acid (PLA) and polyhydroxybutyrate-valerate (PHBV). Natural starch is also frequently offered as a solution, blended with petrochemical-based (mainly potato starch for Bioplast©) or bio-based materials (corn starch with PBAT for Mater-Bi©).

Plastic biodegradation is the microbial conversion of all its organic constituents to carbon dioxide, new microbial biomass and mineral salts under oxic conditions, or to carbon dioxide, methane, new microbial biomass, and mineral salts, under anoxic conditions (Science Advice for Policy by European Academies [SAPEA], 2020). The biodegradability of a plastic is defined according to several standards reflecting disposal condition in soil, compost, or aquatic environments. Tests are generally based on respirometry (biological oxygen demand and CO_2_ production) attributed to the biodegradation of the plastic by microbial communities, without any other source of carbon in artificial medium, in comparison with cellulose, which serves as a reference control (standards ISO 18830:2016, ISO 19679:2016, ASTM D6691-09). Several drawbacks were pointed out by a recent paper on the ability of current standard protocols to realistically predict biodegradability in the marine environment (Harrison et al., 2018). For example, the use of preselected strains does not take into consideration the large diversity of microorganisms colonizing plastics in natural conditions, also known as the “plastisphere” (Zettler et al., 2013) which is still poorly studied. In addition, the conventional techniques used to measure the biodegradation activity by evolution of O_2_ or CO_2_ generally require substantial equipment and can limit the number of experimental replicates. Other activity tests can be considered as alternative methods, such as the use of radiolabeled leucine incorporation that has been used for decades in marine microbiology (Kirchman et al., 1985) or tests based on ATP measurements (Fontanella et al., 2013). When introduced in seawater, plastics are rapidly colonized by bacteria. This process has been extensively studied, showing successive phases of biofilm formation (Dang and Lovell, 2000; Lobelle and Cunliffe, 2011; Eich et al., 2015; Erni-Cassola et al., 2019). During the first days at sea, a “conditioning film” made of inorganic and organic matter supports the initial colonization phase generally initiated by Gammaproteobacteria and Alphaproteobacteria, regardless of the material type (Oberbeckmann et al., 2015). A second phase of rapidly growing bacteria is then usually characterized by a succession of Bacteroidetes, Acidobacteria, Actinobacteria, Cyanobacteria, Firmicutes, and Planctomycetes (Salta et al., 2013). The last phase corresponds to a mature biofilm that generally appears after 15–30 days at sea and remains stable for several months, and where clear differences in bacterial abundance, diversity and activity, were found between non-biodegradable and biodegradable plastics (Eich et al., 2015; Dussud et al., 2018). Hydrocarbonoclastic bacteria (HCB) such as *Alcanivorax* sp., *Aestuariicella hydrocarbonica*, and *Marinobacter* sp. became abundant in the mature biofilm and were suggested to be potentially involved in the degradation of fossil-based plastics. However, no study has extended the experience by incubating the mature biofilm in minimum medium with the plastic as the only carbon source in order to prove its biodegradation capabilities.

Here, we used hollow cylinder sticks, such as those used for cotton swabs, made of different conventional (PP) and “biodegradable” materials as potential candidates for substitutes to conventional single-use plastics (PLA, PBS, PBAT, Mater-Bi, Bioplast, PHBV, and cellulose). We hypothesized that the properties of the distinct materials would select different marine bacterial communities with specific biodegradation capabilities. We elaborated an original two-step experimental design based on a first colonization step in natural seawater (40 days) followed by a second step of incubation in minimum medium with each plastic type as sole source of carbon and energy (94 days). Temporal dynamics of the biofilms associated with each plastic type were followed by measuring bacterial activity (^3^H-leucine incorporation), morphology [scanning electron microscopy (SEM)], and diversity (Illumina sequencing of 16S rRNA genes) during the successive colonization and biodegradation phases.

## Materials and Methods

### Plastic Stick Manufacturing Process

The following list respectively, mentions the material type, its trade name, and the supplier: PP (ISPLEN PP 030 G1E, Repsol), PBAT (Ecoflex C1200, BASF), PLA (Ingeo 7001D, Natureworks), Bioplast (Bioplast 400, Biotec), PHBV (PHBHV ENMAT Y1000P, TianAn Bio), Mater-Bi (Mater-Bi EF04P, Novamont), PBS (bioPBS FZ91PB, PTT MCC Biochem), and cellulose (U Bio). PHBV was preferred in this study because it is a short-chain-length polyhydroxyalkanoate (scl-PHA) that is commercially available. Extended physical and chemical characteristics of the material types are given in Supplementary Table 1.

In order to manufacture plastic sticks that correspond to the hollow cylinder classically used for cotton swabs, we used a machine (except for cellulose sticks) with a spinning line composed of a SCAMEX single screw extruder with a 20-mm diameter and a L/D = 20 ratio. The machine was equipped with a vertical shaping die tube, a cooling tray, a drawing bench, an online dimensional controller (tolerances ± 0.01 mm), and a winding element. In order to conform the plastic materials to the desired stick form, the extruder was equipped with a suitable tube die tailor-made by the extruder manufacturer.

Extrusion conditions were adapted according to the used plastics and to their physical and chemical characteristics (Table 1). Finally, sticks were cut to equivalent dimensions as commercial sticks, i.e., 72-mm length, 2.5-mm outer diameter, and 1.6-mm inner diameter. This study required 300 fragments of 5 mm for each tested material. Except for cellulose, all materials had a hole in the stick.

**TABLE 1:** Extrusion conditions for the different plastic types.

Plastic	Temperature zone 1	Temperature zone 2	Temperature zone 3	Temperature die	Screw speed	Pulling speed
	°C	°C	°C	°C	tr/min	m/min
PP	220	220	220	220	20	3.50
PLA 7001D	170	180	190	180	25	3.90
PHA Y1000P	180	180	180	180	25	4.00
PBAT C1200	140	160	160	160	15	2.90
PBS FZ91PB	140	140	140	140	20	3.50
Mater-Bi EF04P	140	160	160	160	15	3.15
Bioplast 400	160	160	160	160	15	3.00

*PP, polypropylene; PLA, polylactic acid; PHA, polyhydroxyalkanoate; PBAT, polybutylene adipate terephthalate; PBS, poly(butylene succinate).*

### Incubation Under Natural Seawater Conditions (40 Days)

Each material type was incubated in aquariums with direct circulation to the sea, as previously described (Dussud et al., 2018). Briefly, we used nine identical aquariums consisting of trays with a 1.8-L capacity (Sodispan, Madrid, Spain), in which 1.5 L of 200-μm filtered seawater was continually renewed by direct pumping at 14-m depth in the Banyuls Bay, close to the SOLA marine observatory station (42°29′300 N–03°08′700 E, France). A flow rate of 50 ml min^–1^ was chosen to ensure a sufficient renewal of natural bacteria (every 30 min) and a homogeneous distribution of the plastic pieces in the aquariums during the entire experiment.

Approximately 250 pieces of each material type (PP, PLA, PBS, PBAT, Bioplast, Mater-Bi, PHBV, or cellulose) in the form of sticks of 0.5-cm length were washed three times with molecular ethanol and rinsed several times with sterile milliQ water before being placed in the aquariums, with the exception of the “control aquarium,” which only contained the circulating seawater. Pieces were sampled after 7 (D7), 15 (D15), 30 (D30), and 40 days (D40). Aquariums were kept in the dark to avoid UV-driven degradation of the materials. Throughout the incubation, seawater temperature (between 12.5 and 13.5°C) and salinity (38.5) were monitored in the aquariums and were similar to the data found at the SOLA observatory in the Banyuls Bay during this period^1^.

### Incubation in Minimum Medium With Plastic Sticks as Sole Carbon Source (94 Days)

After 40 days under natural seawater conditions, the remaining pieces of each of the eight material types were carefully collected with sterilized tweezers and placed in 4-ml flasks (ref. 294330, Interchim, Montluçon, France) containing 2 ml of minimum medium with no other carbon source than the materials. The composition of the minimum medium was modified from Fegatella et al. (1998): NaCl (420 mM), Na_2_SO_4_ (28.8 mM), KCl (9.39 mM), NaBr (0.84 mM), H_3_BO_3_ (0.485 mM), MgCl_2_⋅6H_2_O (0.546 mM), CaCl_2_ (0.105 mM), NH_4_Cl (9.35 mM), SrCl_2_⋅6H_2_O (0.0638 mM), NaF (0.0714 mM), NaNO_3_ (0.88 mM), NaH_2_PO_4_⋅H_2_O (0.036 mM), KH_2_PO_4_ (0.106 mM), CuSO_4_⋅5H_2_O (0.04 μM), ZnSO_4_⋅7H_2_O (0.08 μM), CoCl_2_⋅6H_2_O (0.04 μM), MnCl_2_⋅4H_2_O (0.9 μM), Na_2_MoO_4_⋅2H_2_O (0.03 μM), FeCl_3_ (1.85 μM), thiamine (0.3 μM), biotin (2.1 nM), and vitamin B12 (0.317 nM). Pieces of each material type were sampled after 3 (D40 + 3), 7 (D40 + 7), 15 (D40 + 15), and 94 days (D40 + 94).

### Scanning Electron Microscopy

Three pieces of each of the eight material types were fixed at the beginning (D0) and at the end of the experiment (D40 + 94) with the addition of 3% (v/v) glutaraldehyde (final concentration) and stored for at least one night at 4°C. SEM was performed as previously described (Goldstein et al., 1992). Briefly, samples were washed in sodium cacodylate buffer and fixed with 1% osmium tetroxide before using successive EtOH baths (70, 95, and 100%) and liquid CO_2_ for dehydration. About 10-nm conductive layer of Au–Pd was then applied on the surface of the samples to allow electron microscopy observation (Hitachi SEM FEG S-4500, Tokyo, Japan).

### Heterotrophic Bacterial Production

Incorporation of ^3^H-leucine is a classical proxy of the bacterial activity in seawater (Kirchman et al., 1985), which has been recently optimized for the plastisphere by Dussud et al. (2018). Bacterial production (BP) was measured on each material type at each sampling time during the colonization and biodegradation phases. A cell detachment pretreatment was applied using three cycles of 3-min sonication bath (Deltasonic, Meaux, France) followed by 3-min vortex at maximum speed (SkyLine, Elmi Ltd., Moscow, Russia). Immediately after cell detachment, ^3^H-leucine (specific activity 123.8 Ci mmol^–1^; PerkinElmer, Waltham, MA, United States) was added to all samples, which consisted of 1.5 ml of sterilized seawater containing the plastic pieces together with the detached cells. Biotic samples were treated in triplicates, and a fourth one was killed with trichloroacetic acid (TCA 50%) and served as a control of the reaction. Abiotic controls (consisting of plastic sticks of the eight material types incubated in sterile seawater) were analyzed following the same protocol. The final leucine concentrations were 36 nmol L^–1^ (^3^H-leucine at 4 nmol L^–1^) during the colonization phase and 150 nmol L^–1^ after the transfer to minimum medium (^3^H-leucine at 1 nmol L^–1^). The saturation of the leucine incorporation was checked during the biodegradation phase, and a higher final leucine concentration was required in order to avoid leucine being limiting (data not shown), explaining why the final leucine concentrations differed between the colonization and degradation phases. The final ^3^H-leucine concentration was 4.3 nmol L^–1^ for control seawater samples. All samples were treated in triplicates and incubated in the dark at *in situ* temperature (between 12 and 15°C) for 2 h. Incubations were stopped by the addition of 50% TCA (5% final concentration), and the plastic sticks or cell pellets were washed with diluted TCA and then with cold ethanol. Radioactivity was counted after addition of liquid scintillator (Filter Count PerkinElmer) on a HIDEX 300 SL scintillation counter (LabLogic Systems, Inc., Tampa, FL, United States).

The empirical conversion factor of 1.55 ng C pmol^–1^ of incorporated leucine was used to calculate BP (Simon and Azam, 1989). Because the exact surface of plastic sticks was difficult to measure (several layers for the cellulose, etc.), each piece was weighted with precision balance of sensitivity 0.1 mg (Precisa 125A, Swiss Quality; Precisa Gravimetrics AG, Dietikon, Switzerland), and the results were expressed in ng C⋅g^–1^⋅h^–1^.

### DNA Extraction, PCR, and Sequencing

For each material type, individual pieces were taken at all sampling times and stored at −80°C until DNA extraction. In parallel, 1 L of seawater was sampled in the control aquarium and successively filtered onto 3- and 0.2-μm pore size polycarbonate filters (47-mm diameter, Nucleopore), and filters were stored at −80°C until analysis. DNA extractions were performed on plastic sticks or filters using a classical phenol–chloroform method (Sauret et al., 2016) with slight modifications of the method for plastic samples (Debeljak et al., 2016). Briefly, the same cell detachment pretreatment was used as for BP (see above) before enzymatic and chemical cell lysis [incubation with 1 mg ml^–1^ of lysozyme at 37°C for 45 min followed by 1 h at 50°C with 0.2 mg ml^–1^ of proteinase K and 1% sodium dodecyl sulfate (SDS)]. DNA was quantified by spectrophotometry (Quant-iT^TM^ PicoGreen^TM^ dsDNA Assay Kit, Invitrogen; Thermo Fisher Scientific, Waltham, MA, United States).

PCR amplification of the V4–V5 region was performed using the primers 515-FY (5′-GTG YCA GCM GCC GCG GTA A-3′) and 926-R (5′-CCG YCA ATT YMT TTR AGT TT-3′) (Fuhrman et al., 1989), which are particularly well suited for marine samples (Parada et al., 2016). Sequencing was performed on Illumina MiSeq by MrDNA (Molecular Research LP, Shallowater, TX, United States) and Integrated Microbiome Resource (IMR; Dalhousie University, Halifax, NS, Canada). The sequences were analyzed using FROGS pipeline hosted in the Galaxy platform (Escudié et al., 2018), following the guidelines given in the publication. Briefly, the forward and reverse reads were assembled; and sequences were clustered using the SWARM algorithm (Mahé et al., 2014) and an aggregation distance of 3. Then, chimeras were removed using the FROGS Vsearch tool (Rognes et al., 2016). An additional filter step was used on the abundance at a 5 × 10^–5^ threshold to select the most relevant operational taxonomic unit (OTU) sequences. The taxonomic affiliation was assigned by standard nucleotid BLAST (Camacho et al., 2009) using the SILVA 132 database 16S (Quast et al., 2013). Chloroplast, mitochondrial, and eukaryotic sequences were removed. All the 16S rRNA data are available in the National Center for Biotechnology Information (NCBI) Sequence Read Archive (SRA) repository (accession number PRJNA649857).

### Statistical Analysis

In order to allow normalization and comparisons of the data, bacterial sequences of each sample were randomly resampled in the OTU file based on the sample with the fewest sequences (*n* = 9,698). These data sets were used to conduct all the analyses performed using the Rstudio software^2^. The α-diversity indices (Chao1, Shannon, evenness, and Simpson) and the β-diversity analysis (the Bray–Curtis similarity) were calculated with the phyloseq package (McMurdie and Holmes, 2013).

Differences between material types were tested using an ANOVA test and confirmed by a *post hoc* Tukey test. Results were considered to be significant if the *p* value was less than 0.05. Dendrogram was obtained based on the dissimilarity matrix of the Bray–Curtis and applying the Ward algorithm (Ward, 1963).

OTUs responsible for dissimilarities between pairs of clusters from the start and the end of incubation in minimum medium were identified using similarity percentage (SIMPER, PRIMER 6).

## Results

### Scanning Electron Microscopy on the Different Plastic Types

**FIGURE 1:**
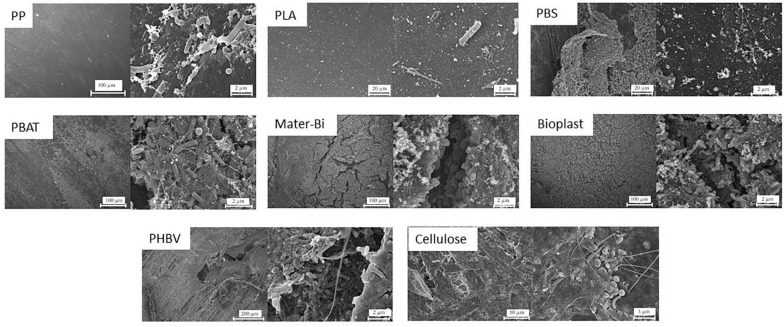
Scanning electron microscopy of the eight polymer types (PP, PLA, PBS, PBAT, Bioplast, Mater-Bi, PHBV, and cellulose) showing the diversity of morphologies and abundance of bacteria cell-likes structures after 40 days in natural seawater followed by 94 days in minimum medium (D40 + 94). PP, polypropylene; PLA, polylactic acid; PBS, poly(butylene succinate); PBAT, polybutylene adipate terephthalate; PHBV, polyhydroxybutyrate-valerate.

Polypropylene, PLA, and PBS surfaces showed no visible signs of biodegradation (cracking, deformation, and holes). In addition, bacterial-cell structures on these materials were scarce and dispersed on the surface after 40 days in natural seawater followed by 94 days in minimum medium (D40 + 94). Abundant microbial colonization was observed on the surface of the other materials (PHBV, Mater-Bi, cellulose, and Bioplast), which is typical of a mature biofilm (Figure 1).

Three-dimensional biofilm structures were visible on the more colonized plastics, where cells colonized not only the surface layers but also the inner part of the visible cracks. A large diversity of morphological forms including spherical, rod-shaped, or spiral-shaped bacterial-like structures were observed on the surface of most of the materials. Extensive exopolysaccharide matrix was found except for PP, PLA, and PBS.

### Bacterial Production During the Successive Phases of Colonization and Transfer to Minimum Medium

**FIGURE 2:**
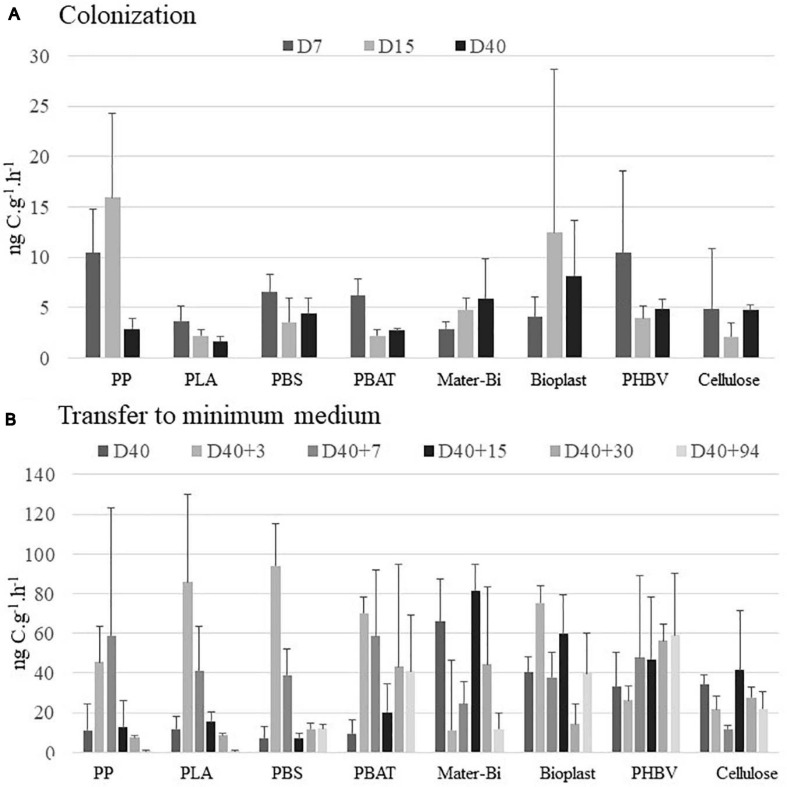
Bacterial production (in ng C⋅g^–1^⋅h^–1^) **(A)** during the colonization phase in natural seawater at days 7 (D7), 15 (D15), and 40 (D40) and **(B)** after the transfer to minimum medium during 3 (D40 + 3), 7 (D40 + 7), 15 (D40 + 15), 30 (D40 + 30), and 94 days (D40 + 94). The vertical bars represent the average of the bacterial production for each material (*n* = 3) ± standard deviation. Changes in BP values at D40 between panels **(A,B)** correspond to changes on dilution factor of the ^3^H-leucine added (see section “Materials and Methods”). BP, bacterial production.

During the colonization phase in natural seawater, BP was generally higher during the growth phase of the biofilm from 7 to 15 days. The highest BP values were found for PP, Bioplast, and PHBV (15.9, 12.4, and 10.5 ng C⋅g^–1^⋅h^–1^, respectively), whereas others (PLA, PBS, PBAT, and cellulose) ranged from 2.2 to 6.5 ng C⋅g^–1^⋅h^–1^ during this colonization phase (Figure 2A). At the end of the colonization phase in natural seawater (D40), BP was even more homogeneous between the material types, ranging from 1.6 to 4.0 ng C⋅g^–1^⋅h^–1^ for PP, PLA, PBS, PBAT and from 4.7 to 8.1 ng C⋅g^–1^⋅h^–1^ for Mater-Bi, Bioplast, PHBV, and cellulose. Bacteria living under control seawater conditions showed stable BP during the 40 days, ranging from 12.4 to 22.3 ng C⋅L^–1^⋅h^–1^ (Supplementary Table 1). No comparison was made between the BP values recorded between the seawater and the plastic sticks because of different measure units.

In order to anticipate the decrease of bacterial heterotrophic activity after the transfer of the plastic together with their biofilms into a minimum medium with the plastic as sole carbon source, we have changed the dilution factor of ^3^H-leucine added to the samples at day 40 accordingly to previous results (data not shown). It did not modify the leucine uptake, but it resulted in an increase of BP values by a factor of 5.8 ± 2.9 (*n* = 8) for all material types. This transfer from natural seawater to minimum medium resulted in different response scenarios according to the plastic types (Figure 2B). A first scenario was found for PP, PLA, and PBS where the BP increased during the first week of incubation in minimum medium (with maxima of 58.8, 85.9, and 94.2 ng C⋅g^–1^⋅h^–1^ at D40 + 3 or D40 + 7) and dramatically decreased thereafter (7.3, 8.7, and 11 ng C⋅g^–1^⋅h^–1^ at D40 + 94). A second scenario was observed for PBAT, Mater-Bi, Bioplast, PHBV, and cellulose, where the BP remained relatively stable after the transfer into minimum medium (41.0, 18.6, 39.8, 59.2, and 21.9 ng C⋅g^–1^⋅h^–1^ at D40 + 94).

### Bacterial Community Structure, Diversity Indices, and Taxonomy During the Successive Phases of Colonization and Transfer to Minimum Medium

A total of 5,427,894 paired-end reads were acquired after sequencing all samples. After normalization based on the sample with the fewest merged sequences (*n* = 9,698), 3,772,255 sequence tags were selected belonging to 978 OTUs (0.03 distance threshold). The seawater control at D40 was sequenced several times, but each sequencing failed for unknown reasons.

#### Changes in α-Diversity Indices

α-Diversity was studied using Chao1 richness, Pielou evenness, and Shannon diversity indices. Regardless of the plastic type, richness rapidly increased during the first 15 days of colonization and reached between 120 and 384 estimated OTUs after 40 days (Supplementary Table 2). The transfer to minimum medium did not influence richness (± 50 estimated species) for some material types including PP, PLA, PBS, and Bioplast but resulted in a decrease for PBAT, Mater-Bi, PHBV, and cellulose when comparing D40 and D40 + 94. We observed that the Chao1 index was statistically linked to the material type after the transfer to minimum medium (ANOVA, *p* value = 0.0147).

The Pielou evenness index showed relatively homogeneous diversity during the colonization phase, with evenness > 0.5 for all plastics after 40 days (Supplementary Table 2). A slight increase was found after the transfer in minimum medium for PP, Bioplast, PHBV, and cellulose, as correlated with the Chao1 index (*p* < 0.05), whereas it slightly decreased for PLA, PBS, PBAT, and Mater-Bi at the end of the incubation.

The resulting Shannon diversity was consistent with the Pielou and Chao1 indices. During the colonization phase, a decrease in the Shannon diversity index was found for the PP (2.4) due to its decrease in evenness (Table 1). A diversity increase was found for all the other plastics at D40 (between 2.64 and 4.40). The transfer to minimum medium resulted in an increase in diversity until the end of the experiment (between 3.67 and 3.9 at D40 + 94) for PP, PHBV, and cellulose according to the increase in evenness. Conversely, a decrease of the Shannon diversity (between 2.1 and 3.64) was caused by a decrease in richness for PBS, PBAT, and Mater-Bi or by a decrease in evenness for PLA and Bioplast.

#### Microbial Communities on Plastics During the Colonization Phase in Seawater

**FIGURE 3:**
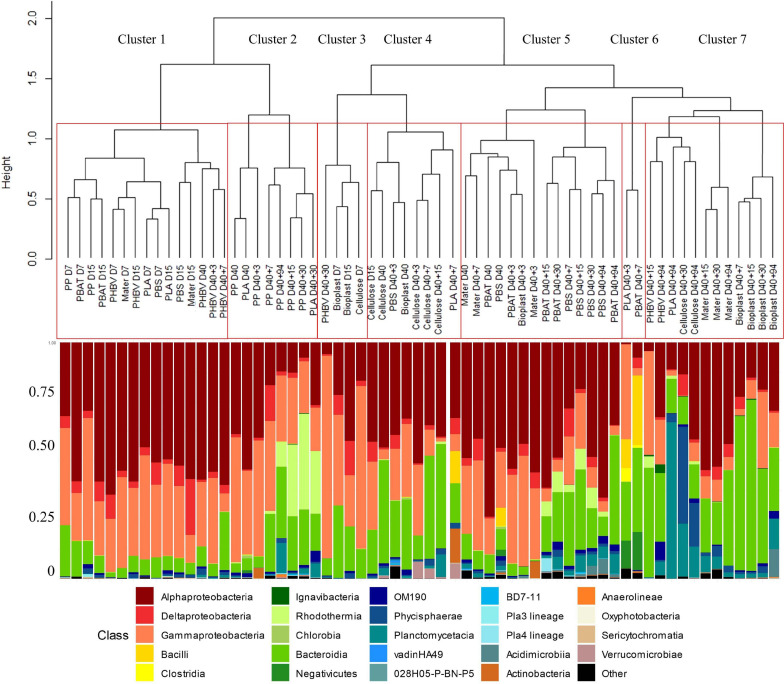
Comparison between hierarchical clustering based on the Bray–Curtis similarity between the temporal dynamic of bacterial communities growing on the eight material types (top part) and their taxonomic affiliation by cumulative charts comparing relative class of abundances (bottom part).

The first colonizers growing on five of the seven plastic types immersed in seawater (PP, PLA, PBS, PBAT, and Mater-Bi) presented very similar communities (D7 and D15 of all material types grouped in Cluster 1) (ANOVA, *p* value = 0.221 and 0.121 for D7 and D15, respectively). Alphaproteobacteria represented 52% of the total OTU abundance in each sample, followed by Gammaproteobacteria (30%) and Bacteroidia (9%). The two most represented families were affiliated to the Alphaproteobacteria, with the Rhodobacteraceae (24%) (mainly *Sulfitobacter* sp. and *Pseudopelagicola* sp.) and the Metyloligellaceae (21%). Flavobacteriaceae (6.7%) (mainly *Croceibacter* sp. and *Aquibacter* sp.) and Nitrinocolaceae (mainly *Neptunibacter* sp.) (5.5%) were also abundant during this stage. Bacteria growing on Bioplast and cellulose were grouped in a separate cluster (Cluster 3, Figure 3), with a lower majority of Alphaproteobacteria (32%) and higher presence of Bacteroidia (25%), together with similar abundance of Gammaproteobacteria (30%) as compared with the other plastic types (see above). Alteromonadaceae family dominated (24.5%) and was mainly represented by the *Alteromonas* sp. and *Marinobacter* sp. Flavobacteriaceae family represented 22% of the biofilm community, mainly represented by the *Wenyingzhuangia* sp. Finally, like the other materials, the Rhodobacteraceae family was very abundant (21%) and was mainly represented by the genus *Sulfitobacter* sp.

Clear changes in bacterial community structures were observed after the growth phase, with clear dissimilarities between the distinct plastic types after 40 days in natural seawater (D40). PP and PLA biofilms (Cluster 2) were dominated by Alphaproteobacteria (Rhodobacteraceae) and Bacteroidia (Flavobacteriaceae). PBS and PBAT (Cluster 5) were dominated by Alphaproteobacteria (mainly Rhodobacteraceae) and Gammaproteobacteria (mainly Solimonadaceae). Bioplast and cellulose (Cluster 4) were dominated by Alphaproteobacteria (mainly Hyphomonadaceae) and Gammaproteobacteria (mainly Alteromonadaceae).

PHBV was dominated by Alphaproteobacteria (Rhodobacteraceae) followed by Gammaproteobacteria (Cellvibrionaceae) and finally Bacteroidia (Flavobacteriaceae). As for Mater-Bi, its mature biofilm was dominated by Alphaproteobacteria (Rhodobacteraceae), followed by Gammaproteobacteria (Spongiibacteraceae) and Bacteroidia (Flavobacteriaceae).

#### Biofilm Specialization After Transfer to Minimum Medium

**FIGURE 4:**
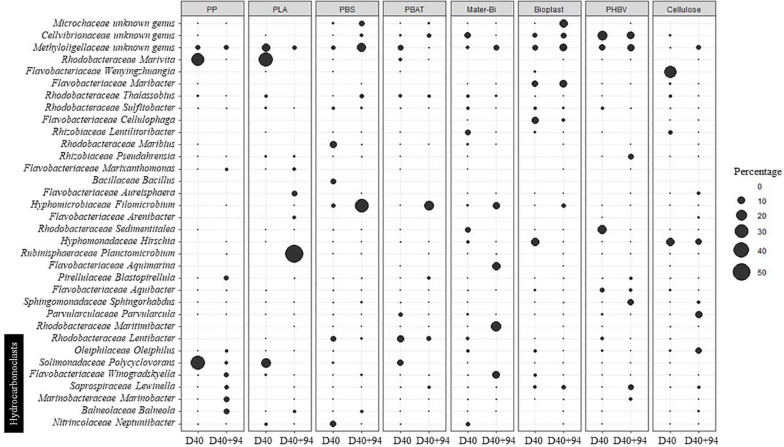
Evolution of the major OTUs (> 4%) on the different materials according to the date of incubation in seawater for 40 days (D40) and in minimum medium for 94 days (D40 + 94). OTUs, operational taxonomic units.

The transfer from natural seawater to minimum medium resulted in bacterial community changes following three different scenarios according to the material types. First, the structures of the bacterial communities associated with PP, PBAT, and PBS remained stable and distinct during the 94 days of incubation. The second scenario was shared between most of the plastic types, where the initial biofilm, established during the colonization phase, remained stable for 1 week after the transfer in minimum medium (D40 + 7) and then switched to another community that remained stable until the end of the experiment (D40 + 94). This scenario was found for Bioplast, Mater-Bi, PHBV, and cellulose, even though bacterial communities growing on each material were grouped in distinct sub-clusters. A third scenario was found for PLA, which presented a chaotic dispersion of the bacterial communities among all other clusters, even if the community at the end of the experiment clustered in the Cluster 7, together with three sub-clusters composed of (i) Mater-Bi, (ii) Bioplast, and (iii) PLA, PHBV, and cellulose. A drastic increase of Bacteroidetes and a decrease of Alpha- and Gammaproteobacteria was observed at the end of the experiment (D40 + 94) in Cluster 7. The evolution of the major OTUs found on each plastic type at the beginning (D40) and at the end (D40 + 94) of the incubation in minimum medium is described in detail in Figure 4.

During seawater incubation, the mature biofilm associated with the non-biodegradable PP and PLA (D40) was largely dominated by *Polycyclovorans* sp. and *Marivita* sp. (more than 62 and 49% for PP and PLA, respectively). These two OTUs became scarce at the end of the incubation in minimum medium (<2.3% at D40 + 94). Bacterial lineages present on PP after 94 days under this drastic condition were hydrocarbonoclasts *Balneola* sp., *Marinobacter* sp., *Winogradskyella* sp., and *Lewinella* sp., explaining 13.15% of the difference between D40 and D40 + 94 and representing 20% (SIMPER analysis) of the relative abundance of the biofilm during the specialization phase. The major OTU at D40 + 94 on PLA was *Planctomicrobium* sp. (56.7% of the community), which contributed to 32.31% of the difference with D40 (SIMPER analysis), where it was not detected. Other genera have emerged at D40 + 94 on this plastic such as *Arenibacter* sp., *Aureisphaera* sp., and *Marixanthomonas* sp. representing almost 11% of the relative abundance at the end of the incubation.

The mature biofilm on PBS (D40) was dominated by *Maribius* sp., *Neptuniibacter* sp., and *Bacillus* sp. representing nearly 21% of the total OTUs, while these three genera represented only 0.64% at D40 + 94. At the end of the experiment (D40 + 94), the biofilm was dominated by *Filomicrobium* sp. (31%), *Thalassobius* sp. (3%), and *Bythopirellula* sp. (2%) explaining about 23% of the disparity with the D40. These three bacterial genera were scarce until transfer into minimum medium (3%).

The dominant OTUs found in the mature biofilm (D40) on PBAT were Rhodobacteraceae at 20.37% (*Marivita* sp., *Lentibacter* sp.), Solimonadaceae at 7.1% (*Lewinella* sp.), and Methylologellaceae at 5.6% and then switched to Saprospiraceae (42.5%) and *Filomicrobium* sp. (15.8%) at D40 + 94. These two OTUs alone explained 34.6% of the difference between the two sampling dates. As already found for PP and PLA, *Polycyclovorans* sp. was very abundant at D40 (7.1%) and was no longer detected at D40 + 94.

Incubation in minimum medium with Mater-Bi as sole carbon source resulted in the selection of *Aquimarina* sp., *Winogradskyella* sp., *Maritimibacter* sp., and *Filomicrobium* sp., which represented nearly 48% of the total OTUs at D40 + 94 but less than 2% at D40. These four genera explained 27.8% of the difference between the two sampling dates based on SIMPER analysis.

The OTUs selected at D40 + 94 for Bioplast were dominated by Microtrichaceae (12%), which belonged to the rare biosphere at D40 (<0.05%). Other genera emerged (> 9%) including *Aliiglaciecola* sp., *Filomicrobium* sp., and *Flexitrhrix* sp., while representing low abundance at D40 (<0.4%). On the contrary, *Alteromonas* sp. and *Oleibacter* sp. were abundant (8.8%) at D40 and became rare at D40 + 94 (<0.05%).

Selection on minimum medium with PHBV as sole carbon source showed the emergence of *Sphingorhabdus* sp., *Lewinella* sp., *Pseudahrensia* sp., and *Marinobacter* sp. (> 21% after D40 + 94 and <0.6% at D40), which explained 17.26% of the difference between D40 and D40 + 94 according to SIMPER analysis (Figure 4), whereas the dominant *Sedimentitalea* sp., *Thalassotalea* sp., and *Lentibacter* sp. at D40 (20% of the total OTUs) represented less than 0.1% at D40 + 94.

Cellulose selected *Parvularcula* sp., *Oleiphilus* sp., *Pirellula* sp., and *Phycisphaera* sp. at D40 + 94 (21.5% of the total OTUs at D40 + 94 compared with <1.5% at D40), which explained 16% of the difference between the two sampling dates. *Hirschia* sp. remained relatively stable between D40 (11.4%) and D40 + 94 (7.32%).

We finally focused on 35 different genera present on all the plastics and that may be considered as potential HCB. In this functional group, Gammaproteobacteria were overrepresented (40% of putative hydrocarbon degraders) followed by Bacteroidia (31%) and Alphaproteobacteria (23%). The most abundant genera potentially involved in hydrocarbon degradation were *Neptuniibacter* sp., *Sulfitobacter* sp., *Balneola* sp., *Lentibacter* sp., and *Marinobacter* sp., which represented 52% of the total OTUs. Overall, putative HCB were abundant during the growth and maturation phases of the biofilms in seawater (between 8.4 and 38.0% of the total communities), with higher abundance generally found during the growth phase for all plastics (Table 2). Interestingly, the putative HCB decreased drastically until the end of the incubation in minimum medium with the plastics as sole carbon sources (D40 + 94). One exception was found for PP, which showed the highest percentage of putative HCB on the mature biofilm (38%), with even higher percentage found after 30 days in minimum medium (63.6%) mainly composed of *Balneola* sp., *Winogradskyella* sp., and *Marinobacter* sp. Other putative HCBs, such as *Polycylovorans* sp., *Neptuniibacter* sp., *Croceibacter* sp., and *Colweillia* sp. (Figure 4), were found in seawater during the colonization phase of most of the materials but did not succeed in colonizing the plastics in minimum medium.

**TABLE 2:** Percentage of OTU selected as putative HCB on each material (PP, PLA, PBS, PBAT, Mater-Bi, Bioplast, PHBV, and cellulose) according to the date of incubation in seawater during 7, 15, and 40 days (D40) followed by the transfer in minimum medium during 3 (40 + 3), 7 (40 + 7), 15 (40 + 15), 30 (40 + 30), and 94 days (40 + 94).

	Natural seawater			Minimum medium				
		
Days	7	15	40	40 + 3	40 + 7	40 + 15	40 + 30	40 + 94
Bioplast	21.1	21.6	12.1	55.1	7.1	9.6	5.8	6.6
Mater-Bi	23.0	14.5	13.3	37.3	19.5	6.2	10.7	13.4
PBAT	31.9	21.3	21.0	54.7	4.8	26.3	19.9	8.6
PBS	21.7	20.7	19.8	12.4	32.7	27.7	22.9	8.6
PHBV	16.0	18.5	8.4	33.4	15.8	8.3	46.1	11.1
PLA	35.1	22.3	22.7	1.4	20.1	NA	35.5	4.9
PP	32.5	26.6	38.0	21.3	37.8	60.2	63.6	26.6
Cellulose	17.4	16.3	11.2	14.1	13.9	3.3	0.9	12.3

*OTU, operational taxonomic unit; HCB, hydrocarbonoclastic bacteria; PP, polypropylene; PLA, polylactic acid; PBS, poly(butylene succinate); PBAT, polybutylene adipate terephthalate; PHBV, polyhydroxybutyrate-valerate.*

## Discussion

### Formation of Mature Biofilms on all Plastic Types in Seawater

#### Aquarium With Direct Circulation to the Sea Mimics the Natural Seawater Conditions of the North Western Mediterranean Sea

The colonization of the different material types was feasible thanks to distinct incubation in aquariums with seawater renewal every 30 min. These aquariums presented similar hydrological conditions to those found at the SOLA observatory in the Banyuls Bay (NW Mediterranean Sea, France). During the first 40 days of incubation in seawater, temperature (13°C ± 0.5°C), chlorophyll *a*, and nutrient values (data not shown) were similar to those usually observed at the long-term SOLA observatory station (Ghiglione et al., 2005; Lambert et al., 2019). Bacterial heterotrophic activity in seawater, measured by ^3^H-leucine incorporation, reached 22.3 ng C⋅L^–1^⋅h^–1^, which was similar to what was generally reported *in situ* in the NW Mediterranean Sea (Pulido-Villena et al., 2012). These results showed that incubation in our aquariums with direct circulation to the sea allowed to reproduce environmental conditions, thus making extrapolation of our results to natural seawater possible.

#### Growth and Maturation Phases of the Biofilms on all the Plastic Types in Seawater

In agreement with patterns observed in another study (Salta et al., 2013), we found a succession of growth and maturation phases of the biofilms for all the material types during the first 40 days of incubation in natural seawater. Each phase was linked with bacterial communities that were similar during the growth phase but became specific to the material types when the mature biofilm formed. This result is in agreement with a previous study conducted in the same region (Dussud et al., 2018).

Bacterial heterotrophic activity peaked during the growth phase of the biofilm in all plastic types (either after 7 or 15 days). These bacterial communities were very similar regardless of the plastic types, except for Bioplast and cellulose, which clustered in another group. This result was surprising considering the distinct chemical material compositions and surface properties of the plastics used in our study. It is noteworthy that bacterial communities growing on Mater-Bi (about 60% corn starch and 40% PBAT) did not cluster with cellulose and Bioplast at this stage (about 40% potato starch and 60% PLA), probably because of different surface properties of the materials that may be related to distinct polymer compositions together with the unknown effect of potentially incorporated additives (not mentioned by the manufacturer). Indeed, early stages of biofilm formation have been depicted as strongly dependent on the surface properties of plastics, including hydrophobicity, structure, and roughness (Lorite et al., 2011). This was not obvious for immersed plastics in seawater, since our results were similar with other studies that did not find distinct communities during the first weeks of biofilm formation on different plastic types (Harrison et al., 2014; De Tender et al., 2017; Dussud et al., 2018). Regardless of the plastic type, the first growth phase was mainly dominated by Proteobacteria (84% of the total abundance on all materials), followed by Bacteroidetes (12.5%). The dominance of Proteobacteria during the first stage of colonization has already been described on the plastisphere (Jacquin et al., 2019). The main classes of the Proteobacteria were Alphaproteobacteria (47.14%, mainly Methyloligellaceae and Rhodobacteraceae) and Gammaproteobacteria (31.46%). The Methyloligellaceae family has already been isolated on other plastic types such as polyamide, polycarbonate, or PP (Feng et al., 2020). All studied plastics were mainly colonized by members of the Rhodobacteraceae during the colonization phase and the specialization phase. This family has been depicted as a key taxonomic group in marine biofilm formation on different material types (Elifantz et al., 2013; Kviatkovski and Minz, 2015; Roager and Sonnenschein, 2019), which explained its large presence on the tested plastics. During the colonization phase, the Flavobacteriaceae family and particularly the genus *Neptuniibacter* sp., generally able to degrade hydrocarbons, was found in abundance on PLA, PBS, and Mater-Bi (Gutierrez, 2019). This result is consistent with a previous study that showed its presence only during the primary colonization phase of plastics under similar conditions (Dussud et al., 2018). In all material types, the mature biofilm that formed after 40 days of incubation in seawater showed lower values of bacterial heterotrophic activity compared with the growth phase. This is consistent with observations made in the unique study measuring bacterial heterotrophic activities on the plastisphere (Dussud et al., 2018). It can be explained by the equilibrium reached by a biofilm at its maturity, which is related to the decrease of available labile inorganic and organic matter and also to the complex interactions (competition, commensalism, etc.) between species coexisting on plastics (Lorite et al., 2011; Henson and Phalak, 2017). Diversity in mature biofilms became specific for each plastic type, which is consistent with observations made in the natural environment (Webb et al., 2012; De Tender et al., 2017; Pinto et al., 2019).

### Convergent Signs of Biodegradation for Some Plastic Types

As recommended in several standard tests (ISO 18830:2016, ISO 19679:2016, and ASTM D6691-09), the biodegradation of the studied plastics was tested after transferring the plastic pieces into minimum medium with no other carbon or energy source (ISO 18830:2016, ISO 19679:2016). As suggested in these tests, we used cellulose as a positive control, and we considered PP as a negative control since its biodegradation was previously depicted to take years or decades in the environment (Barnes et al., 2009). Standards allow the possibility of using two types of inoculum, i.e., artificial mixing of culture of microorganisms or bacterial communities from natural seawater. These methods have been criticized since they are not able to reproduce the mature biofilms usually found on plastics in the environment (Harrison et al., 2018; Jacquin et al., 2019). In our study, we followed the development of a natural mature biofilm on eight different material types (40 days’ incubation in seawater) before using that biofilm as an inoculum for further biodegradation tests.

After 94 days in minimum medium, SEM observations showed no surface modifications for PP, PLA, and PBS, whereas PBAT, Bioplast, Mater-Bi, PHBV, and cellulose exhibited observable morphological alterations, with clear evidence of swelling and erosion. Visible alterations of plastic surfaces are considered to be suitable signs of biodegradation, even if these alterations alone are not sufficient (Zettler et al., 2013). Plastics, such as PHBV, are known to undergo biofragmentation by numerous exo-enzymatic actions (amylases, proteases, and nucleases), leading to weakening of the surface of the material (Deroiné et al., 2014a, 2015). Our results are consistent with a previous study showing evidence of faster degradation of PHAs in seawater compared with other plastics such as PBS, PBAT, or PLA (Sashiwa et al., 2018). In our conditions, PHBV showed more cracks, holes, and colonization features than PBAT, Bioplast, Mater-Bi, PHBV, and cellulose (Figure 1). In parallel, SEM observations at the end of the experiment showed dispersed and isolated bacteria for PP, PLA, and PBS, whereas dense biofilms with a three-dimensional structure and substantial extracellular matrix were found for all the other plastics. PLA was previously found to degrade very slowly in seawater and remain stable at room temperature (Deroiné et al., 2014b), while PBS degradation was found to be slower when salinity increased (Silva Dutra et al., 2019), which are also consistent results with our observations. In our conditions, the radiolabeled leucine incorporation as a proxy of bacterial biodegradation has drawn the same conclusion as the SEM analysis. Interestingly, this is the first time that this technique has been used for this purpose, whereas it has been used for decades to measure the microbial heterotrophic activity and growth in seawater (Kirchman et al., 1985).

It has been successfully used to prove the biodegradability of several other substrates, such as antifouling biocides (Maraldo and Dahllöf, 2004), hydrocarbons (Peng et al., 2020), plasticizers, and flame retardants (Vila-Costa et al., 2019), but never for plastic materials in seawater, probably because of the lack of studies in marine environments. In seawater, this technique is generally preferred to respirometry measurements (O_2_ consumption or CO_2_ production) because its sensitivity is more suitable in oligotrophic waters (Briand et al., 2004; Pulido-Villena et al., 2012). Current standards are based on respirometry analyses and generally measure CO_2_ by gas chromatography, total organic carbon analyzer, infrared analyzer (ISO 14855-1), or CO_2_ capture by soda lime (ISO 14855-2). These experimental approaches can be automated but require a significant financial investment and large laboratory spaces, which can reduce the number of experimental replicates. This has already been described as one of the limitations of the current standards on plastic biodegradation (Harrison et al., 2018). Here, we found that other activity tests based on ^3^H-leucine incorporation were well suited for biodegradation tests of plastic materials in seawater. Our experiments showed that bacteria living in the mature biofilm can continue to be active for several days after transfer under minimum medium with no other carbon source, as it is the case for PP, PLA, and PBS, which showed bacterial heterotrophic activities during 1 or 2 weeks before falling off. Cross-feeding of metabolites synthetized by one species to support the growth of another species to maximize utilization of available resources, together with competition to limited resources, may explain the activity observed during several days and its further decline (Pande et al., 2014; Henson and Phalak, 2017). This is an important point because such an activity could be misinterpreted as a sign of plastic biodegradation if integrative measurements were only taken at the end of the experiment. For PBAT, Bioplast, Mater-Bi, PHBV, and cellulose, the bacterial heterotrophic activity remained rather stable after 15 days in minimum medium and until the end of the experiment (94 days). Taken together with congruent results from SEM observations, we consider that the radiolabeled leucine incorporation measurement was a good proxy of their biodegradation. Our results are consistent with previous findings showing the biodegradation in soil, compost, or seawater for PBAT (Saadi et al., 2013; Weng et al., 2013; Wang et al., 2021), Mater-Bi (Kim et al., 2000), Bioplast^3^, PHBV (Arcos-Hernandez et al., 2015; Deroiné et al., 2015; Wang et al., 2021), and cellulose (Salehpour et al., 2018; Kliem et al., 2020; Zambrano et al., 2020). It is interesting to note that PLA, a plastic considered to be compostable and biodegradable in soil and compost (Ren et al., 2007), did not show any sign of biodegradation under our marine experimental conditions.

### Identification of Putative Biodegraders of the Tested Materials

According to SEM and heterotrophic bacterial activity measurements, we focused on the OTUs selected at the end of the incubation in minimum medium on the plastic materials that showed clear signs of biodegradation under our experimental conditions: PBAT, Bioplast, Mater-Bi, PHBV, and cellulose. The presence of complex communities living under such strict conditions strongly suggest that they are putative biodegraders that may interact with one another in order to perform all or part of the degradation until the ultimate mineralization in CO_2_ (Dussud and Ghiglione, 2014). We are aware that part of the identified OTUs may not be involved in the plastic degradation but rather opportunists that may survive in the biofilm (Sauret et al., 2014). Our original procedure allowed to present here a list of putative OTUs selected from a mature biofilm grown under natural seawater conditions, which may be involved in biodegradation of the plastics.

On the PBAT, we found *Thalassobius* sp. as dominant OTUs after 94 days in minimum medium with the mature biofilm and it’s plastic as sole carbon source. This genus has already been suggested as potentially capable of degrading aromatic hydrocarbons in seawater (Rodrigo-Torres et al., 2017) and found in the oil polluted waters of the Gulf of Mexico (Liu et al., 2017). *Filomicrobium* sp. were also dominant at the end of the experiment and have already been observed in other marine environments (Wu et al., 2009), as well as *Prevotella* sp. and *Streptococcus* sp. known as potential pathogenic genera (Fittipaldi et al., 2012; Larsen, 2017). None of them were previously identified as potential PBAT degraders.

The bacteria growing with Mater-Bi as sole carbon source was dominated by *Filomicrobium* sp., already identified on the plastisphere of PE and PET (Muthukrishnan et al., 2018). Other groups were *Winogradskyella* sp., *Maritimibacter* sp., *Aquimarina* sp., and *Halomonas* sp., which were previously described as putative HCB (Mnif et al., 2009; Liu et al., 2017). None of them were previously identified as potential Mater-Bi (starch with PBAT) degraders.

Bacteria selected on Bioplast at the end of the experiment included *Lewinella* sp., already identified on the plastisphere of fossil-based PP, PE, and PET, as well as on biobased PHBH films (Morohoshi et al., 2018; Roager and Sonnenschein, 2019). Other potential OTUs belonging to putative HCB were *Croceitalea* sp., *Hirschia* sp., *Maribacter* sp., and *Cellulophaga* sp. None of them were previously identified as being potentially able to degrade Bioplast (starch with PLA).

Microbial communities selected after 94 days on PHBV as sole carbon source such as *Marinobacter* sp. and *Alteromonas* sp. were already known for their ability to degrade PHBV (Mergaert et al., 1995; Martinez Tobon, 2019). *Marinobacter* sp. genera contain well-known HCB capable of degrading benzene, toluene, ethylbenzene, and xylene (BTEX) (Li et al., 2012), long-chain alkanes (Grimaud et al., 2012), polycyclic aromatic hydrocarbons (Gao et al., 2013; Cui et al., 2016), chlorinated solvents (Inoue et al., 2020), phenol (Zhou et al., 2020), and PHA (Kasuya et al., 2000; Martinez Tobon, 2019).

Finally, it is noteworthy that putative HCB were less abundant during the biodegradation phase (from 6 to 12% of the total OTUs) than during the colonization phase (from 8 to 35% of the total OTUs). These results were unexpected since several authors proposed that the HCB are certainly involved in plastic biodegradation since they are always found as dominant members of the microbial communities growing on fossil-based plastics recovered from the sea surface (Bryant et al., 2016; Pollet et al., 2018; Amaral-Zettler et al., 2020). Here, we showed that some HCB may actually be involved in biodegradation, but they were not a majority under our experimental conditions. HCB generally present a high ability to attach to particles and to live in a biofilm (Grimaud et al., 2012), two factors that may be more important than their ability to degrade plastics.

### Concluding Remarks

The decisions taken by the European government to reduce the use of single-use plastics and promote biodegradable materials lead to finding substitutes for conventional plastics. Here, we emphasized that materials such as PHBV, Mater-Bi, Bioplast, PBAT, and cellulose are good alternatives to banned cotton swabs generally made of non-biodegradable PP. It is noteworthy that the bio-based polymer PLA showed no signs of biodegradation under our marine conditions, whereas the fossil-based polymer PBAT showed significant evidence of biodegradation. This reinforced the distinction that has to be made between biobased and biodegradable polymers when considering alternative to conventional fossil-based plastics.

Our approach, coupling bacterial diversity and activity measurements, brought new insights on the ability of natural biofilms to biodegrade plastic materials in seawater, with this information being essential for our understanding of the microbial communities involved in the plastisphere and for future alternative biodegradation tests of plastic items in the marine environment. Our study also opens new perspectives by identifying putative bacteria potentially capable of biodegrading the tested plastic materials. These results require further studies to better understand the still largely unexplored functional processes involved in biodegradation in natural seawater.

## Data Availability Statement

The datasets presented in this study can be found in online repositories. The names of the repository/repositories and accession number(s) can be found in the article/Supplementary Material.

## Author Contributions

J-FG, JJ, and NC conceived and designed the study. NC, JJ, CG, JC, and YG carried out all the experiments and acquired the data. SB and CD provided the equipment. NC, JJ, and J-FG analyzed and interpreted the data. JJ, J-FG, NC, CG, and VB drafted the manuscript. MP-P, PC, and A-LM revised the manuscript and approved the version of the manuscript to be published. All authors contributed to the article and approved the submitted version.

## Conflict of Interest

The authors declare that the research was conducted in the absence of any commercial or financial relationships that could be construed as a potential conflict of interest.

## Footnotes

1 https://www.somlit.fr/

2 https://www.r-project.org/

3 https://fr.biotec.de/
