# Supplementary material for: Microbial Diversity and Activity During the Biodegradation in Seawater of Various Substitutes to Conventional Plastic Cotton Swab Sticks
Source: Front Microbiol. 2021 Jul 15;12:604395. doi: 10.3389/fmicb.2021.604395 (PMC8321090; doi:10.3389/fmicb.2021.604395)
Supplement: Supplementary file 1 [file Table_1.docx]

Table S1 : Characteristics of the investigated commercial polymers

| Used acronym | Trade name | Supplier | Origin | T_g_ (°C)^a^ | T_m_ (°C)^b^ | T_c_ (°C)^c^ |
| --- | --- | --- | --- | --- | --- | --- |
| PP | ISPLEN PP 030 G1E | REPSOL | Fossil-based | -10 | 168 | 131 |
| PLA | PLA Ingeo 7001D | NatureWorks | Biobased | 55 | 149 | 122 |
| PHA | PHBHV ENMAT Y1000P | TianAn Biopolymer | Biobased | 5 | 177 | 85 |
| PBAT | Ecoflex F Blend C1200 | BASF | Fossil-based | -30 | 115 | 70 |
| PBS | BioPBS FZ91PB | PTT MCC Biochem | Biobased | -33 | 117 | 90 |
| Mater-Bi | Mater-Bi EF04P | Novamont | Biobased | -29 | 114 | 76 |
| Bioplast | BIOPLAST 400 | Biotec | Biobased | -25 | 124 | 66 |

^a^ Glass transition temperature ^b^ Melting temperature ^C^ Crystallisation temperature
