# Supplementary material for: Microbial Diversity and Activity During the Biodegradation in Seawater of Various Substitutes to Conventional Plastic Cotton Swab Sticks
Source: Front Microbiol. 2021 Jul 15;12:604395. doi: 10.3389/fmicb.2021.604395 (PMC8321090; doi:10.3389/fmicb.2021.604395)
Supplement: Supplementary file 2 [file Table_2.docx]

**Table S2 :** Chao1 richness, Pielou eveness and Shannon diversity indexes for each polymer during a first incubation in seawater during 7, 15 and 40 days followed by the transfer to minimum medium for 3 (40+3), 7 (40+7), 15 (40+15), 30 (40+30) and 94 days (40+94).

| Polymer | Days | OTUs | Chao1 | Pielou | Shannon |
| --- | --- | --- | --- | --- | --- |
| type |  | number | richness | eveness | diversity |
| PP | 7 | 166 | 234 | 0,61 | 3,13 |
|  | 15 | 244 | 295 | 0,69 | 3,82 |
|  | 40 | 204 | 237 | 0,47 | 2,47 |
|  | 40+3 | 108 | 168 | 0,7 | 3,29 |
|  | 40+7 | 201 | 223 | 0,67 | 3,55 |
|  | 40+15 | 186 | 244 | 0,58 | 3,03 |
|  | 40+30 | 208 | 245 | 0,53 | 2,85 |
|  | 40+94 | 212 | 251 | 0,73 | 3,9 |
| PLA | 7 | 144 | 196 | 0,52 | 2,59 |
|  | 15 | 181 | 211 | 0,5 | 2,6 |
|  | 40 | 113 | 120 | 0,56 | 2,64 |
|  | 40+3 | 62 | 67 | 0,61 | 2,5 |
|  | 40+7 | 105 | 144 | 0,72 | 3,35 |
|  | 40+30 | 185 | 214 | 0,64 | 3,34 |
|  | 40+94 | 105 | 121 | 0,45 | 2,1 |
| PBS | 7 | 159 | 196 | 0,6 | 3,05 |
|  | 15 | 126 | 144 | 0,72 | 3,49 |
|  | 40 | 233 | 256 | 0,77 | 4,22 |
|  | 40+3 | 263 | 326 | 0,7 | 3,91 |
|  | 40+7 | 276 | 311 | 0,75 | 4,21 |
|  | 40+15 | 282 | 324 | 0,7 | 3,93 |
|  | 40+30 | 239 | 292 | 0,69 | 3,79 |
|  | 40+94 | 176 | 211 | 0,63 | 3,26 |
| PBAT | 7 | 149 | 194 | 0,58 | 2,91 |
|  | 15 | 231 | 299 | 0,65 | 3,55 |
|  | 40 | 207 | 235 | 0,56 | 2,97 |
|  | 40+3 | 196 | 227 | 0,65 | 3,41 |
|  | 40+7 | 147 | 184 | 0,52 | 2,59 |
|  | 40+15 | 217 | 263 | 0,67 | 3,59 |
|  | 40+30 | 221 | 285 | 0,72 | 3,86 |
|  | 40+94 | 147 | 161 | 0,53 | 2,64 |
| Mater-Bi | 7 | 191 | 238 | 0,57 | 3,01 |
|  | 15 | 116 | 134 | 0,7 | 3,33 |
|  | 40 | 338 | 385 | 0,76 | 4,4 |
|  | 40+3 | 81 | 98 | 0,57 | 2,5 |
|  | 40+7 | 277 | 314 | 0,59 | 3,33 |
|  | 40+15 | 273 | 333 | 0,65 | 3,66 |
|  | 40+30 | 215 | 237 | 0,71 | 3,84 |
|  | 40+94 | 109 | 118 | 0,65 | 3,05 |
| Bioplast | 7 | 138 | 233 | 0,53 | 2,61 |
|  | 15 | 173 | 194 | 0,62 | 3,22 |
|  | 40 | 206 | 221 | 0,75 | 3,98 |
|  | 40+3 | 206 | 255 | 0,55 | 2,92 |
|  | 40+7 | 228 | 262 | 0,61 | 3,31 |
|  | 40+15 | 179 | 202 | 0,57 | 2,97 |
|  | 40+30 | 205 | 234 | 0,67 | 3,57 |
|  | 40+94 | 188 | 241 | 0,69 | 3,64 |
| PHBV | 7 | 157 | 200 | 0,52 | 2,61 |
|  | 15 | 191 | 210 | 0,6 | 3,14 |
|  | 40 | 266 | 303 | 0,6 | 3,37 |
|  | 40+3 | 170 | 202 | 0,58 | 2,97 |
|  | 40+7 | 215 | 240 | 0,66 | 3,56 |
|  | 40+15 | 98 | 117 | 0,36 | 1,66 |
|  | 40+30 | 107 | 128 | 0,39 | 1,83 |
|  | 40+94 | 159 | 175 | 0,72 | 3,67 |
| Cellulose | 7 | 155 | 178 | 0,44 | 2,24 |
|  | 15 | 266 | 349 | 0,71 | 3,98 |
|  | 40 | 275 | 312 | 0,69 | 3,87 |
|  | 40+3 | 192 | 243 | 0,65 | 3,4 |
|  | 40+7 | 236 | 289 | 0,73 | 3,99 |
|  | 40+15 | 195 | 227 | 0,61 | 3,2 |
|  | 40+30 | 171 | 188 | 0,55 | 2,82 |
|  | 40+94 | 199 | 224 | 0,74 | 3,93 |
